# Gastrectomy Versus Esophagectomy for Gastroesophageal Junction Tumors: Short- and Long-Term Outcomes From the Dutch Upper Gastrointestinal Cancer Audit

**DOI:** 10.1097/SLA.0000000000004610

**Published:** 2020-11-17

**Authors:** Egle Jezerskyte, Alexander C. Mertens, Susan van Dieren, Wietse J. Eshuis, Mirjam A. G. Sprangers, Mark I. van Berge Henegouwen, Suzanne S. Gisbertz

**Affiliations:** * Amsterdam UMC, location AMC, University of Amsterdam, Department of Surgery, Cancer Center Amsterdam, Amsterdam, The Netherlands; † Robotics and Mechatronics, University of Twente, Enschede, The Netherlands; ‡ Department of Medical Psychology, Amsterdam UMC, location AMC, University of Amsterdam, The Netherlands

**Keywords:** esophagectomy, esophagogastric junction, gastrectomy, lymph nodes, morbidity, mortality, pathology, survival

## Abstract

Investigate long-term survival, morbidity, mortality, and pathology results in patients following esophagectomy or total gastrectomy for gastroesophageal junction (GEJ) cancer.

**Background:**

Both a total gastrectomy and an esophagectomy may be valid treatment options in patients with GEJ cancer. Which procedure results in the most optimal patient outcome is not well studied. The aim of this study was to investigate the long-term survival, morbidity, mortality, and pathology results in patients following esophagectomy or total gastrectomy for GEJ cancer.

**Methods:**

A retrospective comparative cohort study of prospectively collected data from the Dutch Upper GI Cancer Audit combined with survival data of the Dutch medical insurance database was performed. Patients with GEJ cancer in whom a total gastrectomy or an esophagectomy was performed between 2011 and 2016 were compared. The primary outcome was 3-year overall survival. Postoperative morbidity, mortality, 3-year conditional survival, radicality of resection, and lymph node yield were secondary endpoints.

**Results:**

A total of 871 patients were included: 790 following esophagectomy and 81 following gastrectomy. The 3-year overall survival was 35.8% after esophagectomy and 28.4% after gastrectomy (hazard ratio 1.2, 95% confidence interval 0.721–1.836, *P* = 0.557). Postoperative morbidity, mortality, radicality of resection, lymph node yield, and 3-year conditional survival did not differ significantly between groups.

**Conclusion:**

A total gastrectomy and an esophagectomy for GEJ cancer show largely comparable results with regard to long-term survival, postoperative morbidity, mortality, and pathology results. If both procedures are feasible, other parameters such as surgeon’s experience and quality of life should be considered when planning for surgery.

Cancer of the gastroesophageal junction (GEJ) has a rapidly increasing incidence.^1^ Treatment usually consists of (neo)adjuvant chemo(radio)therapy and surgery.^2^ Both a total gastrectomy and an esophagectomy may be technically possible and selecting the most suitable surgical procedure poses a challenge to surgeons treating GEJ cancer. There is no conclusive evidence which procedure yields the best outcome regarding postoperative morbidity and mortality, pathology results (radicality of surgery and lymph node yield), and long-term survival.

As esophagectomy usually includes a thoracic part, which induces more surgical trauma and, especially if an open esophagectomy is performed, is associated with an increased incidence of pulmonary complications.^3,4^ However, a more extensive lymphadenectomy can be performed during athoracoabdominal approach, compared to an abdominal approach.^5^ Furthermore, a lower rate of R1 resections may be found following a transthoracic approach (esophagectomy) compared to a transhiatal approach (gastrectomy).^6,7^ Two recent systematic reviews reported no difference in 5-year survival, 30-day mortality, and pathology results between esophagectomy and gastrectomy,^8,9^ although one of those systematic review^8^ reported a higher rate of postoperative morbidity after an esophagectomy compared to a gastrectomy. Even though the 30-day mortality is described in most studies included in these systematic reviews, the long-term survival rate is poorly investigated. Also, heterogeneity exists in and between the included studies, as some included not only GEJ but also distal esophageal and gastric cardia cancer, and some excluded patients following neoadjuvant therapy, making results difficult to interpret for patients with true GEJ cancer in the era of neoadjuvant therapy.

The aim of this study was to investigate the difference in longterm survival, postoperative morbidity, mortality, and pathology results in GEJ cancer patients following an esophagectomy or a total gastrectomy at a population level. We hypothesized to find a higher 3-year overall and conditional survival in the esophagectomy group because a more extended lymphadenectomy can be performed with a lower chance of a proximal non-radical resection, however, at the cost of increased postoperative morbidity.

## Methods

### Study Design and Patient Population

The data for this population-based comparative cohort study was obtained from the Dutch Upper Gastrointestinal Cancer Audit (DUCA).^10^ The DUCA is a mandatory national audit, containing prospective data on the diagnostic process and surgical results of all patients with esophageal or gastric cancer operated in the Netherlands. The purpose of this registration is to gain insight into the quality of care and to accelerate its improvement. This system points to potential areas for improvement as hospitals receive feedback on their own results, compared to the national average. Patients in the DUCA are operated by gastrointestinal surgeons, who perform both the thoracic as well as the abdominal part of an esophagectomy. The same surgeons usually also perform the gastrectomies (although in few centers only esophagectomies or only gastrectomies are being performed). Survival data were obtained from VEKTIS, a database of medical insurance organizations of the Netherlands, containing the date of death and information on medical treatments of almost all Dutch people (99%).^11^ Survival data from the VEKTIS database were merged with the DUCA database on the 1^st^ of September, 2017 and the validation of accuracy and completeness has been previously described in a separate article by *van der Werf et al.* ^12^

Surgeons who registered patients in the DUCA database could choose from 10 input options for tumor location: cervical, intrathoracic (proximal part), intrathoracic (middle part), intrathoracic (distal part), esophagus-stomach transition point (GEJ), fundus, corpus, antrum, pylorus and diffuse gastric cancer. Choice for location was made by the responsible surgeon. Patients with an adenocarcinoma of the GEJ were included in this study. Patients who underwent a total gastrectomy or an esophagectomy (transthoracic and transhiatal) with curative intent in the period between January 2011 and December 2016 were compared. Patients in whom no anastomosis was performed or who underwent no resection, patients operated for recurrent disease, or patients with a colonic or jejunal interposition, patients undergoing salvage, palliative or emergency surgery, and patients with a squamous cell carcinoma were excluded from this study. In addition, all patients who underwent a subtotal gastrectomy were excluded. In the Netherlands, a subtotal gastrectomy is a distal gastrectomy, hence cannot be performed for a GEJ cancer. Ethical approval for this study was not required under Dutch law. The STROBE checklist was used for guidance during the composition of this paper.^13^

### (Neo) Adjuvant Therapy

(Neo)adjuvant therapy was administered according to the Dutch guidelines for gastric and esophageal cancer.^14,15^ In case of a true GEJ cancer, patients usually received neoadjuvant chemoradiotherapy according to the CROSS regimen.^16^ Patients with cardia or GEJ cancers extending >2 cm in to the stomach were usually treated with perioperative chemotherapy (EOX: Epirubicin, Oxaliplatin and Capecitabine) according to MAGIC study protocol.^17^ Patients who participated in the CRITICS study received adjuvant chemoradiotherapy following neoadjuvant chemotherapy and gastrectomy.^18^ Patients with World Health Organization functional Classification (WHO) grade ≥3 or early-stage cancer (≤cT2N0) received no neoadjuvant or perioperative therapy.^19^

### Surgical Techniques

Surgery was performed according to the Dutch guidelines for gastric and esophageal cancer.^14,15^ In total gastrectomy, the entire stomach was removed by a minimally invasive or an open approach with a modified D2 lymphadenectomy, after which an esophagoje-junostomy was created with Roux-Y reconstruction. An esophagectomy was either performed open or minimally invasively by a transthoracic (TTE) or transhiatal (THE) approach, with an extended 1-field (THE) or 2-field (TTE) lymphadenectomy, with a cervical or intrathoracic esophagogastric anastomosis.

### Endpoints

The primary endpoint was 3-year overall survival. Long-term disease-specific survival could not be analyzed as the cause of death was not registered in either the VEKTIS or DUCA database. Secondary endpoints were postoperative morbidity [anastomotic dehiscence, pulmonary complications, chyle leakage, cardiac complications, supraventricular arrhythmia, re-interventions, length of Intensive Care Unit (ICU) stay, length of hospital stay, readmissions], short-term mortality (30-day and 90-day), 3-year conditional survival (survival calculated after exclusion of combined 30-day/in-hospital mortality), and pathology results [R0-resection rate, circumferential resection margin (CRM), (positive) lymph node count]. Accurate information on location of resected lymph nodes in the DUCA database is lacking. Since 2016, a division into 5 regions (“intrathoracic high” (paratracheal, laryngeal nerve, aortopulmonal), “intrathoracic low” (subcarinal, paraesophageal), “N1 gastric lymph node stations” (at least 3 of 6), “N2 gastric lymph node stations” (at least 3 of 6), and distant lymph node stations has been added to the registry. As the inclusion period of this study was January 2011 and December 2016, we cannot analyze location of resected lymph nodes in this complete cohort.

### Statistical Analysis

Statistical analysis was performed with SPSS 26.0 software (SPSS, Inc., Chicago, IL). The distribution of continuous variables was assessed using Shapiro-Wilk test. For normally distributed continuous variables, mean values with standard deviation (SD) were reported. In the case of not normally distributed continuous variables, median values with interquartile ranges (IQR) were reported. Binary and categorical variables were reported as proportions. For the analysis of baseline patient and tumor characteristics, Mann-Whitney U test, student t test, *x* ^2^ test, or Fisher exact test was used where applicable. TTE and THE were analyzed separately because results may differ regarding morbidity and lymph node yield. The 3-year overall and conditional survival was displayed using Kaplan Meier survival curves and analyzed using Cox regression analysis. Baseline patient and tumor characteristics with a *P* value <0.1 were added to the multivariable regression analysis as possible confounders using backwards stepwise method. The 3-year overall and conditional survival of patients after TTE, THE, and total gastrectomy was compared to exclude the effect of heterogeneity in the esophagectomy group. Subgroup analyses were performed in patients following perioperative chemotherapy, in patients following neoadjuvant chemoradiotherapy and in (y)pN+ patients. For the analysis of secondary outcomes (postoperative mortality, morbidity, and pathology results), Mann-Whitney U test, student t test, *x* ^2^ test, or Fisher exact test was used where applicable and a Bonferroni correction for multiple testing was performed. If a *P* < 0.1 was found, postoperative morbidity, mortality, and/or pathology results were entered in the multivariable analysis. Multivariable logistic regression was planned for dichotomous variables (postoperative morbidity, re-interventions, mortality, readmissions, and R0 resection rate) and multivariable linear regression was planned for linear variables [length of ICU stay, length of hospital stay, CRM and (positive) lymph node count]. A 2-sided alpha of 0.05 was considered statistically significant.

## Results

### Demographics and Cohort Features

**Figure 1:**
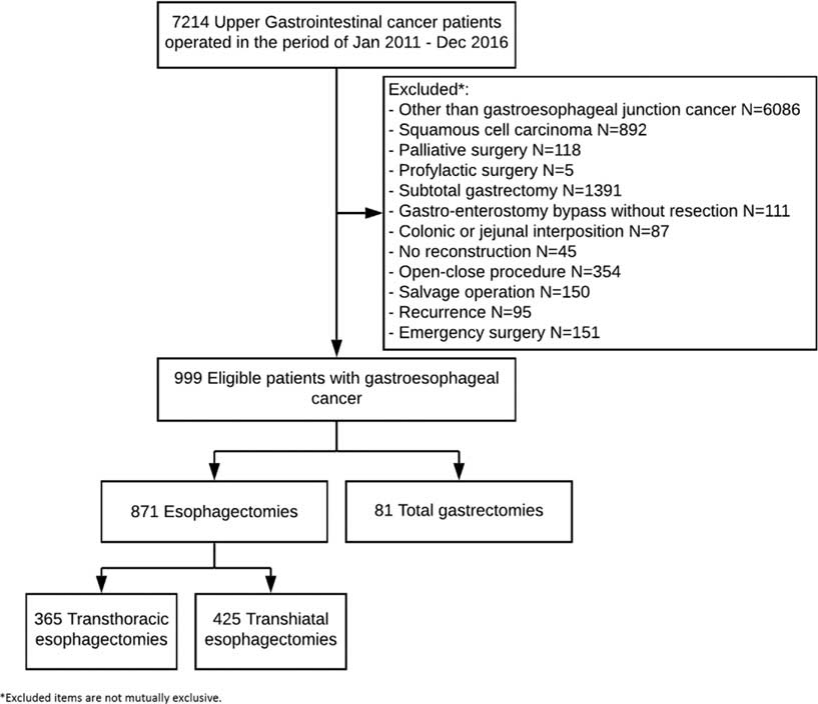
Study flow chart.

A total of 871 patients with GEJ cancer, of 7214 registered upper gastrointestinal (upper GI) cancer patients, were included in the analysis. A total of 790 patients underwent an esophagectomy (365 TTE and 425 THE) and 81 patients underwent a total gastrectomy (Table 1). The reasons for patient exclusion can be found in the flowchart in Figure 1. Most patients were male: 84.4% in the esophagectomy and 82.7% in the gastrectomy group. Patients following an esophagectomy were significantly younger than patients following a gastrectomy [median 65 years (IQR 58–70) vs median 68 years (IQR 60–74), *P* = 0.004]. Patients in the esophagectomy group received significantly more neoadjuvant treatment than patients in the gastrectomy group (92.5% vs 85.1%, *P* < 0.001). The majority of patients receiving neoadjuvant treatment in the gastrectomy group received perioperative chemotherapy (83.8%) and the majority of patients in the esophagectomy group received neoadjuvant chemoradiotherapy (80.1%, *P* < 0.001). An open approach was significantly less common in the esophagectomy group compared to the gastrectomy group (48.2% vs 60.5%, *P* < 0.001).

**Table 1:** Baseline patient and tumor characteristics of patients after transthoracic or transhiatal esophagectomy and total gastrectomy (N = 871) in the period of 2011 –2016

	Transthoracic esophagectomies		Transhiatal esophagectomies		All esophagec-tomies		Gastrectomies		
	N = 365		N = 425		N = 790		N = 81		p value^*^	
Sex (men)	312	(85.5)	355	(83.5)	667	(84.4)	67	(82.7)	0.687
Age, median (IQR), y	64	(57–69)	66	(59–72)	65	(58–70)	68	(60–74)	**0.004**
Body mass index, median (IQR), kg/m^2^	25.0	(23.0–28.0)	25.7	(23.3–29.0)	25.5	(23.1 –28.4)	25.2	(22.7–27.7)	0.238
Comorbidity									
No	124	(34.0)	83	(19.5)	207	(26.2)	19	(23.5)	0.591
Yes	241	(66.0)	342	(80.5)	583	(73.8)	62	(76.5)	
Cardiac	73	(20.0)	116	(27.3)	189	(23.9)	28	(34.6)	**0.035**
Vascular	116	(31.8)	194	(45.6)	310	(39.2)	37	(45.7)	0.260
Diabetic	48	(13.2)	71	(16.7)	119	(15.1)	19	(23.5)	**0.049**
Pulmonary	47	(12.9)	76	(17.9)	123	(15.6)	18	(22.2)	0.122
Thrombotic	13	(3.6)	21	(4.9)	34	(4.3)	12	(14.8)	**0.001**
ASA									
1	82	(22.5)	61	(14.5)	143	(18.2)	10	(12.5)	**0.012**
2	216	(59.2)	249	(59.0)	465	(59.1)	39	(48.8)	
3	67	(18.4)	109	(25.8)	176	(22.4)	30	(37.5)	
4	0	0	3	(0.7)	3	(0.4)	1	(1.3)	
Neoadjuvant therapy									
No	20	(5.5)	39	(9.2)	59	(7.5)	12	(15.0)	**<0.001**
Yes	344	(94.5)	385	(90.8)	729	(92.5)	68	(85.1)	
Chemotherapy	57	(15.6)	88	(20.7)	145	(19.9)	57	(83.8)	**<0.001**
Chemoradiotherapy	287	(78.6)	297	(69.9)	584	(80.1)	11	(16.2)	
cT									
T0	0	0	0	0	0	0	0	0	0.581
T1	13	(3.7)	18	(4.4)	31	(4.1)	3	(4.0)	
T2	50	(14.4)	67	(16.5)	117	(15.5)	16	(21.3)	
T3	275	(79.0)	310	(76.2)	585	(77.5)	55	(73.3)	
T4	10	(2.9)	12	(2.9)	22	(2.9)	1	(1.3)	
cN									
N0	125	(35.8)	154	(38.2)	279	(37.1)	28	(36.8)	0.634
N1	137	(39.3)	168	(41.7)	305	(40.6)	28	(36.8)	
N2	77	(22.1)	70	(17.4)	147	(19.5)	16	(21.1)	
N3	10	(2.9)	11	(2.7)	21	(2.8)	4	(5.3)	
cM									
M0	351	(99.2)	410	(99.5)	761	(99.3)	80	(98.8)	0.454
M1	3	(0.8)	2	(0.5)	5	(0.7)	1	(1.2)	
Approach									
Open	76	(20.8)	305	(71.8)	381	(48.2)	49	(60.5)	**<0.001**
Hybrid	16	(4.4)	115	(27.0)	131	(16.6)	29	(35.8)	
Minimal invasive	273	(74.8)	5	(1.2)	278	(35.2)	3	(3.7)	
Adjuvant therapy									
No	329	(90.6)	363	(86.6)	692	(88.5)	39	(49.4)	**<0.001**
Yes	34	(9.3)	56	(13.2)	90	(11.5)	40	(50.6)	
Chemotherapy	28	(82.4)	51	(91.1)	79	(87.8)	34	(85.0)	0.409
Chemoradiotherapy	6	(17.6)	5	(8.9)	11	(12.2)	5	(12.5)	
Radiotherapy	0	0	0	0	0	0	1	(2.5)	
Histology									
Adenocarcinoma	365	(100)	425	(100)	790	(100)	81	(100)	na
(y)pT									
T0	43	(12.3)	47	(12.1)	90	(12.2)	4	(12.5)	**<0.001**
T1	47	(13.4)	59	(15.1)	106	(14.3)	3	(9.4)	
T2	71	(20.3)	82	(21.0)	153	(20.7)	7	(21.9)	
T3	185	(52.9)	200	(51.3)	385	(52.0)	13	(40.6)	
T4	4	(1.1)	2	(0.5)	6	(0.8)	5	(15.6)	
(y)pN									
N0	182	(51.6)	203	(51.9)	385	(51.7)	15	(46.9)	0.305
N1	80	(22.7)	84	(21.5)	164	(22.0)	6	(18.8)	
N2	55	(15.6)	62	(15.9)	117	(15.7)	4	(12.5)	
N3	36	(10.2)	41	(10.5)	77	(10.3)	7	(21.9)	
(y)pM									
M0	348	(98.9)	405	(98.3)	753	(98.6)	76	(93.8)	**0.013**
M1	4	(1.1)	7	(1.7)	11	(1.4)	5	(6.2)	

Data are presented as n (%) unless otherwise indicated. ASA, American Society of Anesthesiologists; IQR, interquartile range; cTNM indicates clinical TNM staging classification before the treatment (AJCC 8th edition); na, not applicable.

* All esophagectomies vs gastrectomies. Bold *P* values represent significance.

### Primary Endpoint: The 3-Year Overall Survival

**Figure 2:**
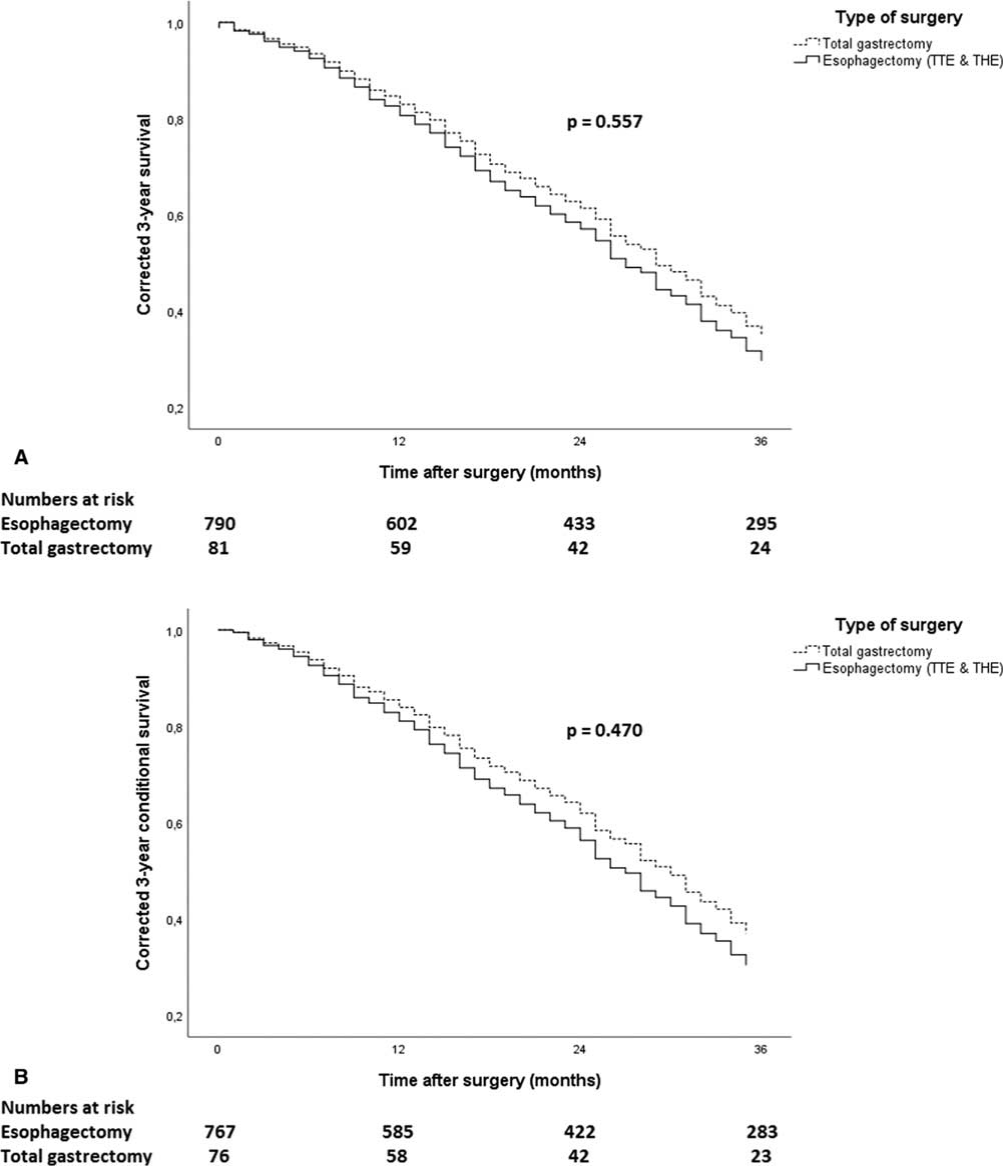
(A) Corrected 3-year overall survival of patients with gastroesophageal junction cancer after an esophagectomy or a total gastrectomy. (B) Corrected 3-year conditional survival of patients with gastroesophageal junction cancer after an esophagectomy.

The Cox proportional hazards assumption was not violated, and Cox regression revealed that the 3-year overall survival was not significantly different between patients undergoing an esophagectomy or a gastrectomy (35.8% vs 28.4%, *P* = 0.557) after correction for the possible confounders age, operation date, comorbidities (cardiac, diabetic, thrombotic), American Society of Anesthesiologists (ASA) classification, neoadjuvant therapy (yes/no and chemoradiotherapy or chemotherapy), surgical approach (open, hybrid, or minimally invasive), adjuvant therapy (yes/no), (y)pT stage, and (y)pM stage (Table 2 and Fig. 2A). The 3-year overall survival did not differ between a TTE, a THE, and a total gastrectomy (supplementary Figure 1 A, http://links.lww.com/SLA/C753). Subgroup analyses in patients following perioperative chemotherapy, in patients following chemoradiotherapy, and in (y)pN+ patients did not show differences in 3-year overall and conditional survival (supplementary Figures 2-4, http://links.lww.com/SLA/C754, http://links.lww.com/SLA/C755, http://links.lww.com/SLA/C756). The number of gastrectomy patients in those subgroup analyses, however, became so small, that strong conclusions cannot be drawn.

**Table 2:** Cox Regression of the 3-year Overall Survival of Patients With Gastroesophageal Cancer After an Esophagectomy or a Total Gastrectomy

		95% CI		
	Hazard Ratio	Lower	Upper	*P*
Esophagectomy	1.2	0.721	1.836	0.557
Age	1.0	0.986	1.007	0.491
Operation date				**<0.001**
2011	1.1	0.735	1.636	0.652
2012	1.4	0.965	2.157	0.074
2013	2.0	1.410	2.929	**<0.001**
2014	5.0	3.456	7.372	**<0.001**
2015	10.0	6.703	15.029	**<0.001**
Cardiac comorbidity	1.0	0.768	1.220	0.782
Diabetes	1.2	0.915	1.520	0.203
Thrombotic comorbidity	1.2	0.786	1.820	0.404
ASA classification				0.372
ASA 1	1.1	0.878	1.454	0.343
ASA 2	1.3	0.962	1.831	0.085
ASA 3	1.1	0.362	3.167	0.902
Neoadjuvant therapy	1.1	0.912	1.285	0.365
Surgical approach	1.0	0.955	1.106	0.468
Adjuvant therapy	0.9	0.655	1.251	0.546
(y)pT stage	1.1	1.068	1.169	**<0.001**
(y)pM stage	1.1	0.602	2.121	0.704

ASA, American Society of Anesthesiologists. Bold *P* values represent significance.

### Secondary Endpoints: 3-Year Conditional Survival, Postoperative Morbidity, Mortality, and Pathology Results

After univariable analysis and correction for multiple testing, no significant difference was found in postoperative morbidity, 30-day and 90-day mortality, length of hospital stay, readmissions, reinterventions, positive lymph node count, R0 resection rate, or median CRM between the esophagectomy and gastrectomy groups (Table 3). However, after univariable analysis a difference with a *P* < 0.1 between esophagectomy and gastrectomy groups was found in length of ICU stay [median 2 days (IQR 1 –4) vs median 1 day (IQR 0–2), *P* < 0.001] and total lymph node count [median 17 (IQR 12–23) vs median 21 (IQR 16-31), *P* < 0.001]. A multivariable linear regression was performed including a correction for the possible confounders age, operation date, comorbidities (cardiac, diabetic, thrombotic), ASA classification, neoadjuvant therapy (yes/no and chemoradiotherapy or chemotherapy), surgical approach (open, hybrid or minimally invasive), adjuvant therapy (yes/no), (y)pT stage, and/or (y)pM stage. However, no significant difference was found between esophagectomy and gastrectomy in length of ICU stay [ß = 3.6, 95% confidence interval (CI) –0.043 to 7.292, P = 0.053] or total lymph node count (ß = -3.1,95% CI –6.446 to 0.165, *P* = 0.063) (Table 4). In addition, no significant difference was found in 3-year conditional survival between patients following an esophagectomy or a gastrectomy (36.9% vs 30.3%, *P* = 0.470) (Table 5 and Fig. 2B). The 3-year conditional survival was also not significantly different between a TTE, a THE, and a total gastrectomy (supplementary Figure 1 B, http://links.lww.com/SLA/C753).

**Table 3:** Secondary Endpoints (Postoperative Morbidity, Mortality and Pathology Results) of 871 Patients With Gastroesophageal Cancer After Esophagectomy or Total Gastrectomy

	Transthoracic Esophagectomies		Transhiatal Esophagectomies		All Esophagectomies		Gastrectomies			
	N = 365		N = 425		N = 790		N = 81		*P* ^*^	Corrected *P*
Postoperative morbidity										
No	157	(43.0)	194	(46.2)	351	(44.7)	41	(50.6)	0.309	6.180
Yes	208	(57.0)	226	(53.8)	434	(55.3)	40	(49.4)		
Anastomotic leakage	58	(17.6)	83	(20.1)	14	(18.2)	14	(18.2)	0.861	17.220
Pulmonary complications	104	(28.5)	104	(24.8)	208	(26.5)	23	(28.4)	0.718	14.360
Chyle leakage	37	(10.3)	4	(1.0)	41	(5.3)	3	(3.8)	0.790	15.800
Cardiac complications	37	(10.1)	48	(11.5)	85	(10.8)	23	(28.4)	0.541	10.820
Supraventricular arrhythmia	7	(4.2)	4	(2.0)	11	(3.0)	1	(2.3)	0.801	16.020
Other	37	(10.1)	36	(8.6)	73	(9.3)	7	(8.6)	0.843	16.860
Re-interventions										
Yes	94	(25.8)	71	(16.9)	165	(21.0)	25	(31.3)	**0.036**	0.720
Radiologic	37	(39.8)	31	(45.6)	68	(42.2)	14	(56.0)	0.197	3.940
Endoscopic	38	(40.9)	11	(16.4)	49	(30.6)	8	(32.0)	0.890	17.800
Re-operation	55	(58.5)	48	(69.6)	103	(63.2)	14	(56.0)	0.490	9.800
Length of ICU stay, median (IQR), days	2	(1–4)	1	(1–3)	2	(1–4)	1	(0–2)	**<0.001**	**<0.001**
Length of hospital stay, median (IQR), days	12	(9–19)	11	(9–16)	12	(9–17)	10	(8–19)	0.094	1.880
Readmissions	58	(16.1)	48	(11.4)	106	(13.4)	12	(14.8)	0.687	13.740
30-day/In-hospital mortality	6	(1.6)	13	(3.1)	19	(2.4)	3	(3.7)	0.450	9.000
90-day Mortality	6	(1.6)	10	(2.4)	16	(2.0)	2	(2.5)	0.680	13.600
Resection rate										
R0	335	(92.3)	337	(90.0)	712	(91.0)	71	(87.7)	0.316	6.320
R1	28	(7.7)	39	(9.3)	67	(8.6)	10	(12.3)		
R2	0	0	3	(0.7)	3	(0.4)	0	0		
Circumferential resection margin (median [IQR], mm)	4	(2–8)	3	(1–6.5)	3	(1–7)	3.5	(1–10)	0.956	19.120
Total lymph node count, median (IQR)	20	(15–27)	15	(10–20)	17	(12–23)	21	(16–31)	**<0.001**	**<0.001**
Positive lymph node count, median (IQR)	0	(0–3)	0	(0–3)	0	(0–3)	1	(0–5)	0.138	2.760

Data are presented as n (%) unless otherwise indicated. ICU, Intensive Care Unit; IQR, interquartile range. Bold *P* values represent significance.

* All esophagectomies vs gastrectomies.

**Table 4:** Multivariable Linear Regression Analysis of Lymph Node Count and Length of ICU Stay of Patients With Gastroesophageal Cancer After Esophagectomy or Total Gastrectomy

		95% CI		
Covariates	B	Lower	Upper	*P* ^*^
Lymph node count				
Esophagectomy	–3.1	–6.446	0.165	0.063
Age	–0.1	–0.198	–0.062	**<0.001**
Operation date	0.7	0.283	1.108	**0.001**
Cardiac comorbidity	—	—	—	—
Diabetes	—	—	—	—
Thrombotic comorbidity	—	—	—	—
ASA classification	—	—	—	—
Neoadjuvant therapy	–1.9	–3.018	–0.857	**<0.001**
Surgical approach	2.1	1.555	2.563	**<0.001**
Adjuvant therapy	2.2	0.055	4.273	**0.044**
(y)pT stage	0.3	0.041	0.611	**0.025**
(y)pM stage	–6.4	–11.150	–1.661	**0.008**
Length of ICU stay				
Esophagectomy	3.6	–0.043	7.292	0.053
Age	—	—	—	—
Operation date	—	—	—	—
Cardiac comorbidity	—	—	—	—
Diabetes	—	—	—	—
Thrombotic comorbidity	—	—	—	—
ASA classification	2.0	0.861	3.132	**0.001**
Neoadjuvant therapy	–0.7	–1.982	0.494	0.239
Surgical approach	0.3	–0.186	0.873	0.204
Adjuvant therapy	–1.2	–3.579	1.248	0.343
(y)pT stage	–0.2	–0.481	0.166	0.338
(y)pM stage	—	—	—	—

ASA, American Society of Anesthesiologists; B indicates regression coefficient.

* All esophagectomies vs gastrectomies. Bold *P* values represent significance.

**Table 5:** Cox Regression of the 3-year Conditional Survival of Patients With Gastroesophageal Cancer After an Esopha-gectomy or a Total Gastrectomy

		95% CI		
	Hazard Ratio	Lower Upper	Upper	*P*
Esophagectomy	1.2	0.735	1.951	0.470
Age	1.0	0.985	1.006	0.366
Operation date				**<0.001**
2011	1.1	0.697	1.590	0.806
2012	1.4	0.914	2.092	0.125
2013	2.1	1.439	3.031	**<0.001**
2014	5.1	3.454	7.514	**<0.001**
2015	10.9	7.189	16.468	**<0.001**
Cardiac comorbidity	0.9	0.726	1.174	0.517
Diabetes	1.1	0.881	1.494	0.309
Thrombotic comorbidity	1.2	0.793	1.870	0.369
ASA classification				0.569
ASA 1	1.1	0.868	1.442	0.387
ASA 2	1.3	0.913	1.761	0.157
ASA 3	1.2	0.392	3.480	0.780
Neoadjuvant therapy	1.1	0.911	1.300	0.350
Surgical approach	1.0	0.966	1.122	0.290
Adjuvant therapy	1.0	0.694	1.332	0.813
(y)pT stage	1.1	1.072	1.177	**<0.001**
(y)pM stage	1.1	0.554	2.072	0.837

Bold *P* values represent significance.

ASA, American Society of Anesthesiologists. Bold *P* values represent significanc.

## Discussion

This study investigated the long-term survival, postoperative morbidity, mortality, and pathology results in patients following an esophagectomy or a total gastrectomy for GEJ cancer. The results show that the 3-year overall and conditional survival of patients with GEJ cancer undergoing either an esophagectomy or a gastrectomy did not differ significantly. In addition, postoperative morbidity, short-term mortality, and pathology results were not different between the 2 surgical approaches. Although not in line with our hypothesis, the study results contribute to clinical decision making by showing that both procedures can be performed with comparable short- and long-term results for patients with GEJ cancer. In addition, a recent study showed that the majority of patients have significant symptoms >1 year following an esophagectomy^20^; however, largely comparable results were found between a gastrectomy and an esophagectomy regarding long-term quality of life.^21^ Therefore, surgeons can base their decision with respect to the operative procedure for patients with GEJ cancer on personal and center experience.

A high heterogeneity was observed across previous studies investigating long-term survival, morbidity, mortality, and pathology results in patients following an esophagectomy or a total gastrectomy.^8,9^ In some studies, administration of neoadjuvant therapy was either excluded or not reported and most studies also included distal esophageal or cardia/gastric cancers, rendering the comparison with our results difficult. Nonetheless, 2 systematic reviews, mostly comprised of retrospective mono-center studies, reported no significant difference in 5-year overall survival, 30-day mortality, lymph node yield, or radicality of surgery, although a higher rate of postoperative morbidity was seen after esophagectomy in one. The most recent systematic review included 1 randomized controlled trial (RCT).^22^ This RCT compared patients after left thoracoabdominal approach (N = 85) versus abdominal-transhiatal approach (N = 82) and found no significant difference in 5-year overall survival but increased morbidity after left thoracoabdominal approach. In this RCT, only patients with (sub)cardia cancer were included and all patients were operated by an open approach via a left thoracoabdominal incision, and therefore, the results of this RCT cannot be directly compared to the present study. Furthermore, a recent study with the national audit data (DUCA) compared the quality of the surgical resection, morbidity and mortality between a TTE and a THE esophagectomy in patients with a distal esophageal or GEJ cancer.^23^ In this study, an increased morbidity and short-term mortality was found in the transthoracic group, but also a higher lymph node yield. However, long-term survival was not investigated. In the present study, a subgroup analysis of 3-year overall and conditional survival between TTE and THE was performed and showed no significant difference between the 2 groups. However, these results are only applicable to patients with GEJ cancer.

In the Netherlands, centralization of esophageal cancer surgery was initiated in 2011, whereas centralization of gastric cancer surgery was initiated 2 years later, in 2013. Since then, a minimum of 20 esophagectomies and 20 gastrectomies yearly is required to perform either gastrectomies or esophagectomies at a center. Mortality rates for gastrectomy have dropped from 7.7% in 2011 to 4.4% in 2018, whereas mortality rates following esophagectomy have dropped as well, but were already much lower compared to gastrectomy (4.1% in 2011 and 2.7% in 2018).^2,24^ This delayed centralization for gastrectomies may partially explain why a higher morbidity following gastrectomy was observed in this population-based study. In addition, the pathology results, with a high R0 resection and similar (y)pN0 rate in both groups probably contribute to the comparable survival rates in both groups, even though a higher (y)pT4 rate was observed in the gastrectomy group.

Furthermore, minimally invasive esophagectomy is associated with less pulmonary complications compared to open esophagectomy.^25^ Minimally invasive esophagectomy has become the preferred approach in the Netherlands, where 90.9% of all esophagectomies were performed minimally invasively in 2018.^2^ Also, in this study the majority of patients were operated minimally invasively, perhaps that is why less postoperative morbidity than expected was observed in the esophagectomy group. The comparable postoperative morbidity results may also contribute to similar longterm survival, as has been shown that survival may impair in patients with severe complications.^12^

Various retrospective studies have been unable to determine the optimal extent of lymph node dissection for GEJ cancer.^26–28^ A recent prospective study investigated the incidence of lymph node metastases in each lymph node station in patients with a GEJ tumor. They found a >10% rate of lymph node metastases in stations 1, 2, 3, 7, 9, and 11p, and at least 1 of the lower mediastinal lymph node stations. If esophageal involvement exceeded 4 cm, station 106recR (right recurrent laryngeal nerve) was also affected in >10% of the cases and other upper middle and lower mediastinal lymph node stations were regularly affected. Therefore, the authors propose to perform a right transthoracic approach in all patients with a GEJ tumor that invades the esophagus for >4 cm.^29^ Unfortunately, such detailed information in the DUCA database is lacking. The registration of the location of resected lymph nodes in the DUCA database started in 2016 and the location of lymph node metastases is not recorded. Therefore, we could only provide information about whether a lymphadenectomy was performed. Due to a thoracic as well as an abdominal phase of the surgery we expected more lymph nodes to be resected in patients who underwent an esophagectomy. We found no significant difference in total lymph node count however, between patients who underwent an esophagectomy or a gastrectomy. Also, there was no significant difference in positive lymph node count or radicality of surgery. Our findings are in accordance with a recent systematic review where also no difference in total lymph node count and R0-resection rate was found between esophagectomy and gastrectomy in patients with GEJ cancer.^8^

A large difference in the number of patients with GEJ cancer treated with either an esophagectomy or a gastrectomy is seen in our data. Apparently, in the Netherlands, a preference for an esophagectomy exists for patients with GEJ cancer, although the reasons for this selection are unknown. This preference could be based on tumor characteristics (eg, slightly more ingrowth in the distal esophagus), on surgeon’s experience, or expert opinion; there are, however, no data to support this.

The present study has several limitations. It is a retrospective comparative cohort study of prospectively collected data and no propensity score matching could be performed as it would have highly reduced the number of included patients with GEJ cancer who underwent a total gastrectomy. Furthermore, the DUCA-VEKTIS database was merged on the 1st of September 2017, and therefore no survival data after this date were available. Also, no disease-specific survival could be analyzed as the cause of death is neither reported in the DUCA nor VEKTIS database. Since DUCA only recently added the Clavien-Dindo classification for postoperative complications to the audit, these data were unavailable for the vast majority of our cohort, and as such could not be analyzed. The anatomical location of the GEJ cancer could not be classified according to the Siewert-Stein classification,^30^ as it is not included in the DUCA database. However, extensive input options for esophageal tumor location are available in the DUCA database, including cervical, proximal intrathoracic, midthoracic, distal thoracic, and esophagus-stomach transition point and this choice is made by the responsible surgeon. The number of statistical tests performed was high; therefore, a Bonferroni correction for multiple testing was performed, to counteract the possibility of finding a significant difference by chance. Furthermore, this study does not include any patient-reported outcome measures.

A strength of the current multicenter study in the Netherlands is that it includes one of the largest samples of patients with GEJ cancer with long-term survival data at a population level.

In conclusion, this study shows that an esophagectomy and a total gastrectomy in patients with GEJ cancer show largely comparable results with regard to postoperative morbidity and mortality, pathology results, as well as long-term survival. Other parameters such as surgeon’s experience should be considered when planning surgery if both procedures are technically feasible. However, these results need confirmation by RCTs.

## Supplementary Material

SUPPLEMENTARY MATERIAL
