# Supplementary material for: Gastrectomy Versus Esophagectomy for Gastroesophageal Junction Tumors: Short- and Long-Term Outcomes From the Dutch Upper Gastrointestinal Cancer Audit
Source: Ann Surg. 2020 Nov 17;276(6):e735–43. doi: 10.1097/SLA.0000000000004610 (PMC9645547; doi:10.1097/SLA.0000000000004610)
Supplement: SUPPLEMENTARY MATERIAL [file sla-276-e735-s001.docx]

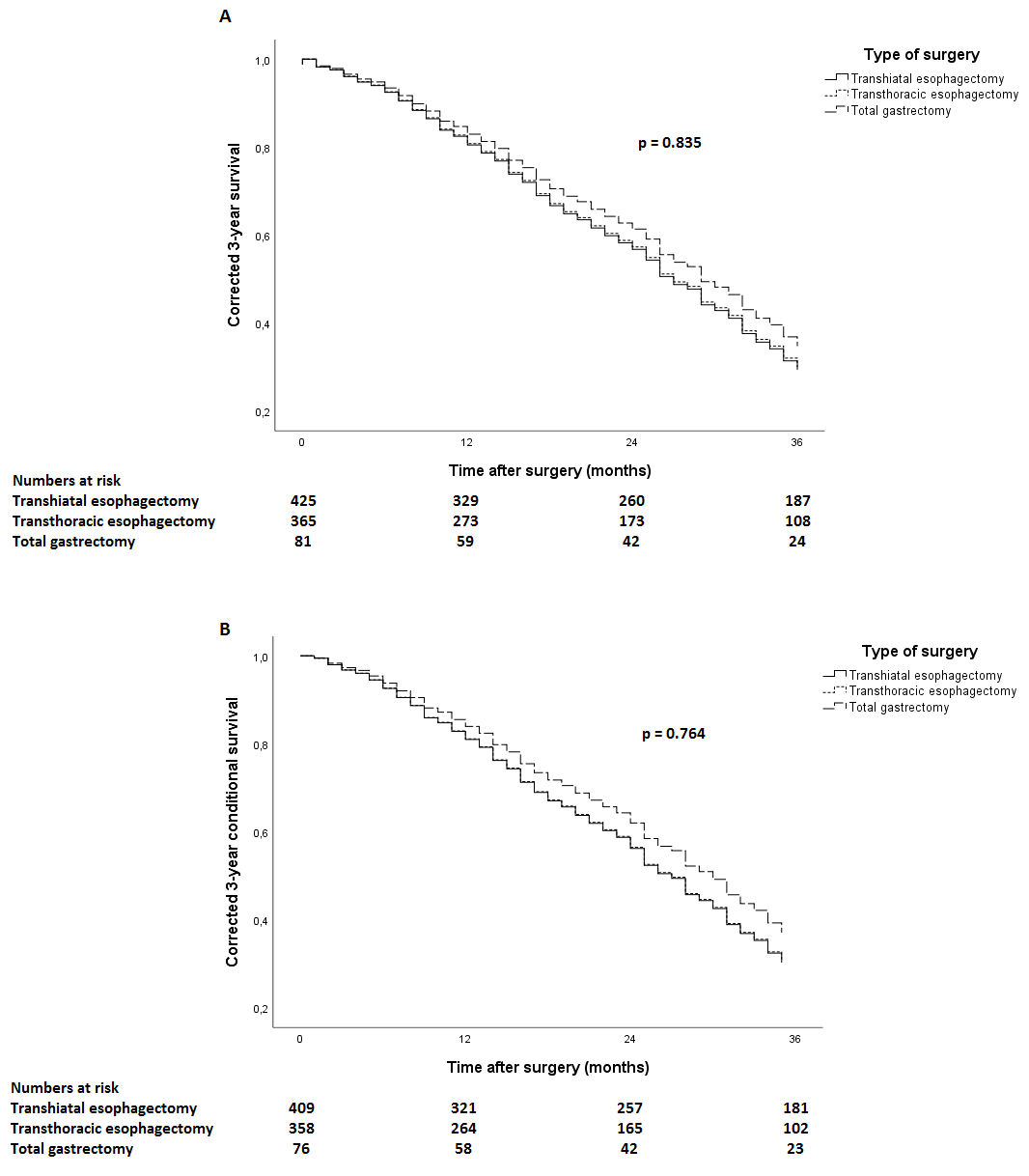


*Supplementary figure 1 A & B:*_A: corrected 3-year overall survival of patients with gastroesophageal junction cancer after transhiatal esophagectomy, transthoracic esophagectomy or total gastrectomy. B: corrected 3-year conditional survival of patients with gastroesophageal junction cancer after transhiatal esophagectomy, transthoracic esophagectomy or total gastrectomy_
